# Burden of Disease and Unmet Needs in the Diagnosis and Management of Atopic Dermatitis in Korea

**DOI:** 10.3390/jcm12113744

**Published:** 2023-05-29

**Authors:** Yu Ri Woo, Hei Sung Kim

**Affiliations:** Department of Dermatology, Incheon St. Mary’s Hospital, College of Medicine, The Catholic University of Korea, Seoul 06591, Republic of Korea

**Keywords:** atopic dermatitis, management, quality of life, diagnosis, treatment, Korea

## Abstract

Atopic dermatitis (AD) is a chronic inflammatory skin condition associated with a significant disease burden in Korea. AD is highly prevalent among Korean children, adolescents, and adults, and can cause physical discomfort, psychological distress, and social isolation for those affected. Despite advances in our understanding of AD, there are still many unmet needs in diagnosing and managing the disease in Korea. One of the challenges in diagnosing AD is the lack of a definitive biomarker for AD in Korea, and there is a need for more effective, safe, and cost-effective treatments for AD. Therefore, finding out the current epidemiology, burden of AD, and how AD is currently being diagnosed in Korea and reviewing management options available in Korea will help resolve the unmet needs of AD patients in Korea. Addressing these and other unmet needs in AD management and diagnosis in Korea may improve outcomes for those affected by this challenging condition.

## 1. Introduction

Atopic dermatitis (AD), also known as atopic eczema, is a common chronic inflammatory skin disease affecting people of all ages. Pruritus is one of the primary features of AD [1,2] and caused by various factors, including impairment of the epidermal barrier, dysregulation of T helper cells, keratinocyte-derived proinflammatory cytokines, various pruritogens, and interactions with cutaneous nerve fibers [3]. Among various symptoms of AD, pruritus was the most bothersome symptom in patients with AD [4]. and therefore significantly impact the quality of life (QoL) of those affected [2].

AD poses a substantial public health burden due to its high prevalence and significant patient burden [5]. Figuring out the exact epidemiology of AD is challenging due to the heterogeneity of clinical characteristics, various diagnostic criteria for AD, and the absence of high-quality biomarkers for diagnosing AD [6,7,8]. Clinical characteristics of AD differ by ethnicity, and management options available vary from country to country. Therefore, identifying the burden of AD and unmet needs in management and diagnosis in Korea is vital for several reasons. Identifying unmet needs in AD can help healthcare providers and policymakers allocate resources more effectively to address the needs of those affected by AD. Determining gaps in current management options and diagnostic strategies can help improve patient outcomes by developing more effective interventions. Therefore, this review aims to address the epidemiology of AD in Korea and the psychological burden of Korean AD patients. In addition, finding out how AD is currently being diagnosed in Korea and reviewing management options available in Korea will help resolve the unmet needs of AD patients in Korea.

## 2. Materials and Methods

We searched the PubMed, Google Scholar, and Embase databases for this review. The literature search was conducted using the following terms to retrieve relevant articles including “atopic dermatitis”, “atopic eczema”, “Korea”, “epidemiology”, “quality of life”, “clinical”, “diagnosis”, “comorbidity’, “education”, “treatment”, “management”, “dupilumab”, “abrocitinib”, “baricitinib”, or “upadacitinib”. The search terms were tailored for each electronic database to maintain consistency. We included English-language articles and Korean-language articles published between 1993 to 2023. We only included the original articles and review papers on AD. Studies only with abstracts or conference posters were excluded. An additional literature search was conducted to identify further studies considered relevant in the above databases.

## 3. Epidemiology of AD in Korea

Although many studies have determined the prevalence of AD, it is difficult to compare the results from various studies due to discrepancies in study designs and definitions of AD. The global prevalence of AD is estimated to be around 15–20% in children and 1–3% in adults [9]. The prevalence of AD varies significantly depending on various factors such as age, geography, genetics, and lifestyle. As for childhood, one of the most extensive global estimates of AD prevalence was determined by the International Study of Asthma and Allergies in Childhood (ISAAC) by utilizing the United Kingdom Working Party criteria for the definition of AD [10]. According to the ISAAC study, the worldwide prevalence of AD in adults varies from country to country ranging from 0.9% to 22.5% at ages 6 to 7 years old and 0.2% to 24.6% at ages 13 to 14 years old [5]. In the United States, a study by Laughter et al. [11] reported that AD prevalence of Oregon schoolchildren aged 5 to 9 years was 17.2% by self-administrated questionnaire and 11.8% by the question “Has a doctor ever said that your child has eczema?”. The prevalence estimates of adult AD in the United States also vary from the study, which ranges from 3.2% to 10.2% due to differences in the definitions of AD [12,13].

In Korea, the estimated prevalence of AD varies from study to study. In one recent study, the estimated prevalence of AD was reported as 5.9% in infants (<2 years), 11.3% in preschool children (2–5 years), and 14.6% in school-age children (6–18 years), 3.9% in adults (19–59 years), and 1.6% in the elderly (≥60 years), respectively, based on the Korean National Health and Nutrition Examination Survey (KNHANES) among the 2016 to 2017 population [14]. KNHANES is a representative health information survey conducted by the Korea Disease Control and Prevention Agency (KDCA). The authors also reported a significant increase in the estimated prevalence of AD in school-aged children and the elderly over 10 years (from 2008 to 2017) [14]. In addition, the estimated prevalence of AD in infants and preschool children tends to be decreased over 10 years (from 2008 to 2017) [14]. Although these divergent trends cannot be explained clearly to date, the authors suggest that social and environmental changes might contribute to a change in this trend [14]. In another study, the estimated prevalence of AD in children under 18 was reported as 13.50% using data from 2008–2011 KNHANES [15].

Among adults who underwent health examinations in Korea in 2009, the prevalence of AD was reported at 7.1% when surveyed by questionnaire and 2.6% when examined by a doctor, indicating a higher prevalence rate of AD through the questionnaire based examination [16]. The experience rate for doctor diagnosis of AD by KNHANES for Koreans aged older than 19 increased from 2.9% in 2009 to 5.2% in 2020 [17]. Based on the findings from previous studies, the prevalence of AD in Korea is estimated to be about 10–15% in children and then gradually decreases with age to about 3–5% in adults.

It is important to note that the prevalence of AD can vary depending on the population studied, the diagnostic criteria used, and the methodologies of each study, as well as other factors. However, overall, AD is a relatively common condition that affects a significant portion of the population in Korea.

## 4. Psychological Burden of AD in Korea

AD has a chronic, relapsing disease course. Indeed, patients experience repeated exacerbations and improvements during their clinical course [18,19]. The QoL of patients with AD can vary greatly depending on various factors, such as the severity of their symptoms and exposed skin areas [20,21,22,23]. To date, various types of research have been conducted to identify the QoL of Korean patients with AD (Table 1).

There are various tools for assessing the QoL among adult AD patients. When utilizing the Dermatology Life Quality Index (DLQI), the mean DLQI for adult Korean AD was 10.7 (standard deviation [SD], 7.9) in a hospital-based cross-sectional study [24]. There was no gender difference concerning the DLQI score. However, adults with atopic comorbidities, including concomitant asthma, allergic rhinitis, or allergic conjunctivitis, showed higher DLQI (mean ± SD, 11.7 ± 8.0) than those with AD alone (mean ± SD, 9.7 ± 7.8) [24]. A recent study including 1163 Korean AD patients aged over 19 years found that 72.3% of the patients experienced a moderate to severe impact on their QoL (DLQI score: 6–30) [25]. That study included 10.9% of the patients with severe AD (eczema area severity index [EASI] ≥ 21) and found that the severity of DLQI was positively associated with AD severity (EASI) [25]. They also reported that skin lesions in any functionally important or visible area, including perioral, eyelids, genital, palm, and soles, are associated with social and functional impairment, inducing a low QoL [25].

As for children, the study by Seong et al. [26] found the mean score for children’s DLQI (CDLQI) of Korean school-aged children aged 7–19 years was 8.04 ± 6.29, and this was positively correlated with the SCORing of Atopic Dermatitis (SCORAD) index. A study by Park et al. [27] also reported that the QoL of children aged 0 to 6 years was 11.50 ± 6.2 by infants’ dermatologic quality of life (IDQoL). The top three highest scores for the items of IDQoL were observed in association with itching and scratching, problems with bath time, and mood [27]. The study by Cho et al. [28] reported that the mean CDLQI in Korean children and adolescents with AD aged 7 to 18 years was 12.83 ± 6.52. In that study, the CDLQI showed a positive correlation with EASI scores [28], suggesting that the severity of AD correlated with decrements in QoL of children and adolescent patients with AD. The items most affected by the QoL of children and adolescents with AD were symptoms such as itching, and the least affected item was interpersonal relationships, which is consistent with the results of other studies [29,30]. In other studies conducted outside of Korea, the mean value of CDLQI in children was observed in the range of 6.0 to 9.73 [29,31]. The relatively high CDLQI observed in Korean AD children in some studies is probably related to selection bias because those studies were primarily conducted on patients visiting a tertiary hospital.

To determine the effect of AD on the patient’s families, a study by Park et al. [27] used the Dermatitis Family Impact (DFI) questionnaire, which ranges from 0–30, and the higher the sum of DFI, the more significant the negative impact on the family of AD patients. The mean DFI of Korean child AD patients’ families was 11.2 ± 6.0. The logistic regression analysis was performed to predict the dependent factors associated with the parent’s QoL [27]. The parents with a strong negative emotionality had a 3.8 times higher probability and parents with girls with AD had an 8 times higher probability; parents with severe AD had a 6.6 times higher probability of experiencing low QoL than parents of children with those who did not [27].

The burden of AD is significant and can cause psychological distress. AD symptoms, including intense itch, have been known to be a pivotal factor in the vicious cycle of AD [32]. In addition, due to the unfavorable appearance of AD skin lesions, AD patients frequently feel anxiety, particularly during social activities with other people [32]. Indeed, many studies have confirmed that AD is associated with various psychological conditions such as depression, anxiety, stress, sleep disturbance, and suicidal ideation [33,34]. Studies have shown that AD can have a considerable psychological burden on patients in Korea.

A nationwide population-based cross-sectional study to identify the psychological burden of Korean AD patients found that Korean adult patients with AD showed significant severe psychological stress, an increased prevalence of depressed mood and use of psychological counseling services, and an increased prevalence of depression and suicidal ideation when compared to non-AD individuals [35]. When utilizing the Euro QoL (EQ) 5-dimension (EQ-5D) questionnaire to identify the health-related QoL (HRQoL) among Koreans, increased rates of having some or severe pain/discomfort (EQ-4) and anxiety/depression (EQ-5) on the EQ-5D was observed in patients with AD when compared to individuals without AD [35]. A recent nationwide population-based cross-sectional study using the KNHANES reported that adult-onset AD patients showed increased odds of having higher EQ-5D (for physical activity, self-control, daily activities, pain/discomfort, and anxiety/depression) than healthy controls [36]. However, patients with child-onset AD only showed significantly increased odds only in pain/discomfort among the EQ-5D questionnaires [36]. The authors suggested that adult-onset AD patients suffer significantly poorer QoL than normal controls [36]. The study by Son et al. [25] also suggested that Korean adults AD patients exhibited increased odds of having stress (odds ratio [OR], 1.74; 95% confidence interval [CI], 1.14–2.65), depression (OR, 1.69; 95% CI, 1.00–2.84), and suicidal ideation (OR, 1.66; 95% CI, 1.02–2.69). The severe AD patients showed higher rates of development of suicidal ideation or attempt (35.5%) and diagnosis of depression or anxiety (16.7%) [25].

**Table 1:** Summary of studies that measured health-related quality of life in Korean atopic dermatitis patients.

Authors	Participants	Tools Used for AD Severity	Tools Used for QoL	Outcomes Measures	Main Outcomes
Cho et al. [28]	46 children and adolescents with AD aged 7–18 years (mean age, 11.2)	CDLQI	EASI	The mean score of CDLQI and EASI was 12.8 and 8.51, respectively.	The CDLQI and EASI scores showed a significant correlation. Among the subcategories of CDLQI, sleep, itching, and clothes showed a high correlation with the EASI score.
Kim et al. [37]	82 patients and 47 parents (mean age, 19.7)	SCORAD	A questionnaire designed explicitly by the authors’ clinical experience	The mean QoL score was 30.1. The SCORAD for each patient was as follows: mild <20, n = 25; moderate 20–40, n = 39; severe >40, n = 40.	The QoL scores significantly positively correlated with the severity of AD.
Park et al. [27]	101 children with AD	EASI	IDQOL, DFI, FI	The mean scores for IDQOL, DFI, FI, and EASI were 11.50, 10.05, 16.10, and 8.8, respectively.	The mean score of EASI was positively correlated with the IDQOL, DFI, and FI, respectively.
Seong et al. [26]	78 AD patients aged 7–19 years (mean age, 14.1)	SCORAD	CDLQI, CDI, K-ARS	The mean score for SCORAD and CDLQI were 28.95 and 8.04, respectively.	The CDLQI was significantly correlated with the SCORAD. Psychiatric symptoms (CDI and K-ARS) did not show a correlation with the SCORAD, but they were correlated with the CDLQI score.
Kim et al. [38]	31 adults AD aged 19–43 years (mean age, 27.2)	EASI	DLQI, WHOQOL-BREF	The mean DLQI, QOL, and EASI scores were 19.74, 3.77, and 9.42, respectively.	DLQI and WHOQOL-BREF scores significantly correlated with the EASI score. Some subitems for DLQI (emotion, daily life, work, and school work) showed a high correlation with the EASI score.
Kong et al. [39]	50 adults AD (mean age, 26.4) and 50 children AD patients (mean age, 4.9)	SCORAD	CDLQI, DLQI CSHQ, PSQI	The mean scores of SCORAD, DLQI, and PSQI in adult were 34.8, 10.67, and 8.51, respectively; the mean scores of SCORAD, CDLQI, and CSHQ in children were 26.76, 9.31, and 42.67, respectively.	The SCORAD and CSHQ score, the SCORAD and CDLQI score, and the CSHQ and CDLQI score showed significant correlations. Significant correlations existed between the SCORAD and DLQI scores and the PSQI and DLQI scores.
Lee et al. [35]	677 adults with AD (mean age, 36.1) and 36,901 control (mean age, 45.4)	N/A	EQ-5D, EQ-VAS	The mean scores for EQ-5D subcomponents in AD and control were as follows: EQ-1, 10.4 and 12.8; EQ-2, 3.1 and 3.6, EQ-3, 7.0 and 8.2; EQ-4, 27.1 and 21.5; EQ-5, 16.0 and 10.9). The mean EQ-VAS score in AD and normal was 71.8 and 74.3, respectively.	Patients with AD showed higher rates of severe pain/discomfort (EQ-4) and anxiety/depression (EQ-5) on EQ-5D than healthy controls. Patients with AD showed higher EQ-VAS than those without AD.
Yoo et al. [36]	383 child-onset AD patients (mean age, 26.57), 440 adult-onset AD patients (mean age, 43.54), and 32,806 normal (mean age, 44.98)	N/A	EQ-5D	N/A	The increased odds ratio of EQ-5D questionnaire responses was observed in adult-onset AD compared to normal controls.
Son et al. [25]	1163 adult AD patients aged >19 years (mean age, 31.6)	EASI	DLQI	The score of EASI was categorized into mild (47.1%), moderate (42.0%), and severe (10.9%), respectively. The moderate to severe impact on QoL (DLQI 6–30) was observed in 72.3%, and a no-to-small impact on QoL (0–5) was observed in 27.7%.	DLQI severity was significantly associated with EASI.

Abbreviations: CDI, Kovacs’ Children’s Depression Inventory; CDLQI, children’s dermatology life quality index; CSHQ, children’s sleep habits questionnaire; DFI, Dermatitis Family Impact questionnaire; EASI, eczema area and severity index; EQ-5D, EuroQoL 5-dimension questionnaire; EQ-VAS, EQ-visual analogue scale; FI, Financial Impact questionnaire; IDQOL, Infants’ Dermatologic Quality of Life Index; K-ARS, Korean Attention-Deficit Hyperactivity Disorder Rating Scale; N/A, not accessible; PSQI, Pittsburgh sleep quality index; QoL, quality of life; SCORAD, SCORing atopic dermatitis; WHOQOL-BREF, World Health Organization QOL assessment instrument-brief version.

A cross-sectional survey to identify the psychological burden of Korean adolescents with AD encompassing 15,536 adolescents with AD reported that Korean adolescents with AD showed an increased rate of stress (59.1%), depression (27.8%), and suicidal ideation (13.9%) compared to non-AD individuals [40]. Depression and suicidal ideation were reciprocally essential factors in adolescents with AD [40]. In that study, the male sex was a risk factor for influencing stress, depression, and suicidal ideation [40]. The authors hypothesized that uncontrolled impulsivity and not expressing their emotions frequently among adolescent males might influence this observation [40]. In a study based on the 2013 Korean Youth risk behavior survey among adolescent middle and high school students in Korea, adolescents with AD showed statistically significant increased odds of experiencing suicidal ideation (OR, 1.26; 95% CI, 1.16–1.36), suicide planning (OR, 1.28; 95% CI, 1.14–1.44), and suicide attempt (OR, 1.29; 95% CI, 1.13–1.49) [41].

In addition, Kong et al. [39] reported that Korean children and adults with AD patients suffer from poor sleep quality by the children’s sleep habits questionnaire (CSHQ). Among CSHQ items, AD severity (SCORAD) was significantly associated with bedtime resistance, sleep onset delay, sleep anxiety, parasomnias, and sleep-disordered breathing [39]. In addition, the severity of AD (SCORAD) was positively correlated with CSHQ and CDLQI scores, respectively [39]. Moreover, CSHQ significantly correlated with the CDLQI score [39].

There are several measures to evaluate health related-QoL and psychological burdens, and it is difficult to directly compare the results of various studies because the tools used in the study are diverse, and the severity of patients included in the studies varies. However, when reviewing various studies, the QoLs of adult, adolescent, and pediatric AD patients and their parents have deteriorated. The clinical severity of AD is correlated with poor QoL in most patients with AD. The social stigma associated with AD in Korea can also contribute to the psychological burden of the condition. In particular, skin lesions in the visible sites and functionally important areas can be associated with shame and embarrassment in patients’ daily lives. To decrease the psychological burden of AD in Korea, healthcare providers recommend various interventions, including psychological counseling, support groups, and stress management techniques. They may also provide education and resources to help patients and their families cope with the condition and improve their QoL. Further detailed interventions that have been implemented in Korea will be discussed later. However, despite these efforts, patients are still adversely affected by the QoL due to AD, especially in patients with severe disease. Therefore, the latest effective and cost-effective treatment options that can reduce the severity of AD must also be provided for these patients.

## 5. Clinical Features and Diagnostic Criteria of AD in Korea

Clinical manifestations of AD vary and can be observed in various ways depending on race and age as well as individual differences. Since specific diagnostic biomarkers related to AD have not been established, Hanifin et al. [42] suggested 4 main and 23 minor findings according to the patient’s medical history and clinical features in 1980. Although many countries have since diagnosed AD using Hanifin and Rajka’s criteria (HRC), the criteria have limitations for universal use by all races worldwide, so each country has created and used several slightly modified diagnostic criteria.

The characteristic clinical features of Korean patients with AD have been confirmed through several studies. The characteristic minor features in Korean pediatric AD patients include dry skin, scalp scaling, forehead lichenification, periauricular eczema, infragluteal eczema, perifollicular accentuation, periorbital eczema, and eczema on the hands and feet [43,44,45,46]. Park et al. [43] compared the characteristic minor features observed in Korean pediatric AD patients with normal controls. Among 38 minor features, dry skin, scalp scaling, forehead lichenification, white dermographisms, eyelid eczema, Dennie–Morgan wrinkles, pityriasis alba, periauricular fissure/eczema, elbow/knee/malleolar sandpaper-like skin lesions, ventral wrist eczema, and infragluteal eczema were identified as statistically significant minor features [43]. According to the study by Kim et al. [47], dry skin, itching when sweating, skin lesions around the eyes, nonspecific eczema on the hands and feet, and hypersensitivity to wool were commonly observed minor features in Korean pediatric patients with AD symptoms. Although not included in HRC, five additional features of AD, including scalp scaling, postauricular fissure, infra-auricular fissure, forehead lichenification, and infragluteal eczema, were found to be significantly more frequently observed in Korean pediatric AD patients [47].

The common skin symptoms in adult Korean patients with AD include dry skin, white dermographism, scalp scaling, forehead lichenification, Hertoghe’s sign, eyelid eczema, orbital darkening, Dennie–Morgan fold, pityriasis alba, auricular fissure/eczema, cheilitis, facial erythema/pallor, anterior neck fold, hyperlinear palm, perifollicular accentuation, ichthyosis-like skin lesion, sandpaper-like skin lesion, nipple eczema, ventral wrist eczema, infragluteal eczema, nummular eczema, and pompholyx [43,48]. Lindmaier et al. [49] suggested dandruff as a symptom of AD because it showed a low specificity but high sensitivity in diagnosing AD. In addition, many studies have reported the diffuse scaling of the scalp as a minor feature of AD [50,51], a finding also commonly observed in Korean AD patients [48]. The diagnostic criteria of AD in Korea, which modified the HRC considering the characteristic clinical features of Korean AD, were announced in 2005 by the Korean Atopic Dermatitis Association (Table 2) [52]. AD diagnosis can be made in Korea when at least 2 of the 4 major features and 4 of 14 minor features are fulfilled. These criteria have been widely used in clinical practice and studies conducted on Korean AD patients [53,54,55].

Recently, the simplified 2-plus-1 AD diagnostic criteria, which comprise two essential features and one important feature, is also recommended by the American Academy of Dermatology (AAD) [56]. Moreover, the Japanese Dermatological Association also suggested that patients having three basic items, including pruritus, typical morphology and distribution of eczema, and the chronic or chronically relapsing course, could be diagnosed with AD regardless of the severity of symptoms [57]. A trend toward simplifying the diagnostic criteria for AD is observed worldwide. The Korean Atopic Dermatitis Association (KADA) is also preparing to update the diagnostic criteria for AD in Korea, and the updated diagnostic criteria of AD in Korea will be announced soon.

Regarding allergic comorbidities, 44% of AD patients reported having a personal history of allergic comorbidities [58]. A study by Chu et al. [59] recently determined the allergic comorbidities in Korean patients with AD. A total of 34.4% of AD patients had allergic comorbidities, including allergic rhinitis, asthma, and allergic conjunctivitis [59]. In their analysis, 78.3% of Korean AD patients with allergic comorbidities have a single comorbidity, whereas the rest showed multiple allergic comorbid conditions [59]. Among allergic comorbidities, allergic rhinitis was the most common (82.0%), followed by allergic conjunctivitis (10.9%) and asthma (7.1%) [59].

Among several allergens, the highest rates of sensitization were observed in *D. farinae* (59.7%) and *D. pteronyssinus* (59.5%) among Korean AD patients by using immunoassay CAP in one study [59]. Another study determining the allergen sensitization in Korean patients with AD found the highest sensitization rates in house dust mites and house dust in adult and children AD patients by using multiple allergen simultaneous test (MAST) [60]. Since there are few studies on these points in Korea, identifying allergens associated with the severity of AD requires further research in Korea.

## 6. Management Options of AD in Korea

Following AD diagnosis, proper management of AD should be conducted based on the treatment guidelines. The guidelines for AD treatment are continuously updated to introduce newer treatment options for AD and to provide the up-to-date risk–benefit of systemic immunosuppressants and advanced therapies.

Based on the Korean treatment guidelines for AD, all patients should use moisturizers regularly [61]. Moisturizer helps alleviate pruritus and has short- and long-term steroid-sparing effects [61]. In addition, bathing can help manage AD by providing skin hydration and removing crust and irritants. Regular bathing is recommended in Korea for managing AD patients. Generally, using a non-soap cleanser is essential for bathing with warm or lukewarm water for a short period [61]. Moreover, identifying and limiting exposure to possible triggering factors, such as animal dander, house dust mite, wool, sweat, personal hygiene products, foods, exercise, perfumes, stress, and alcohol, are recommended [61]. Such triggering factors can be differed by patients’ age, social lifestyles, and environment [61]. However, the elimination of specific foods should be performed only when an allergy to the specific food is confirmed by proper methods, including an allergy test by a specialist [61].

Educational interventions and counseling for patients with AD and caregivers are also recommended for successful treatment in updated consensus guidelines of AD in Korea [61]. To provide effective educational interventions, some suggest using a multidisciplinary team approach [62]. However, such a multidisciplinary team approach is not usually active in general clinical practice in Korea. In this regard, institutional improvements such as economic support are needed. One study reported that a half-day, a family-engaged educational program that was held every year since 2005 in Korea, was helping in improving knowledge about AD in children with AD and their parents, improving parents’ management skills for AD, and positively impacting the course of the disease and the family’s QoL [63].

According to the clinical severity of AD, patients with mild AD assessed by objective SCORAD < 15 or EASI < 16 can be treated with topical calcineurin inhibitors, topical steroids, and antihistamines for active treatment of AD in Korea [64]. Although antihistamine is not usually recommended in treating AD in some countries, Korean experts still recommend using antihistamines to control pruritus in AD [64]. They suggest using antihistamines to help relieve pruritus and prevent exacerbation associated with patients’ scratching behavior [64].

In patients with moderate to severe AD, defined as having an objective EASI score of ≥16, in addition to topical steroids, topical calcineurin inhibitors, and antihistamines, short-term use of systemic steroids, cyclosporine, immunomodulatory drugs including azathioprine, methotrexate, and mycophenolate mofetil, as well as biologics (dupilumab), are currently considered treatment options in Korea treatment guidelines [65]. 

Among various biologics approved for AD in other countries, only dupilumab is currently approved by the Ministry of Food and Drug Safety of the Republic of Korea (KFDA) in AD. Dupilumab is a fully human IL-4Rα monoclonal antibody, which inhibits both IL-4 and IL-13 signaling in AD. Several clinical studies have confirmed the clinical effectiveness and safety of dupilumab [66,67,68]. In Korea, dupilumab was approved in March 2018 for treating AD in adults aged 18 years and older who were not adequately controlled with topical therapies or for which these therapies are not recommended [65]. It expanded its indication to adolescents aged 12 to 17 years in April 2020, to children aged 6 years to 11 years in March 2021, and to children aged 6 months to 5 years in November 2022. A retrospective real-world study investigating the efficacy and safety of dupilumab in Korean patients with moderate to severe AD found that patients achieving EASI 50, EASI 75, EASI 90, and a change of at least four points in the DLQI score (minimal clinically important difference, MCID) were achieved after 6 months of treatment with dupilumab in 84.6%, 61.5%, 26.9%, and 91.7% of patients, respectively [69]. Another real-world study identifying the long-term effectiveness and safety of dupilumab in Korean patients with AD reported that patients achieving EASI 75 and 90 were reported as 90.2% and 53.7%, respectively, after 52 weeks of treatment with dupilumab [70], which is a slightly higher rate of EASI improvement than previous studies [71,72,73]. The authors suggest that relatively more remarkable improvement of EASI after dupilumab treatment in Korean patients may be due to the advantages of the Korean healthcare system, including easy access to hospitals, which allows patients to visit doctors more frequently, and thorough education on the use of topical calcineurin inhibitors as a proactive treatment of AD [70]. 

Recently, Jak inhibitors (upadacitinib, abrocitinib, baricitinib) have been approved for treating patients with AD by the KFDA and are currently considered treatment options for AD in Korea. The baricitinib, a selective JAK1 and JAK2 inhibitor, is the first JAK inhibitor approved by KFDA in May 2021 for treating adults with moderate to severe AD in Korea. The treatment with baricitinib showed significantly improved symptoms and signs of moderate to severe AD in several phase 3 clinical trials [74,75,76,77]. Upadacitinib, a selective JAK 1 inhibitor, was approved for its use by the KFDA in October 2021 to treat moderate to severe AD in patients aged 12 years and older in Korea. 

A real-world retrospective study determining the efficacy and safety of upadacitinib in Korean patients with AD found that patients achieving EASI 75, EASI 90, and EASI 100 were reported as 61.54%, 30.77%, and 15.38%, respectively, after 16 weeks of treatment with upadacitinib 15 mg [78]. The most common side effect after treatment with upadacitinib was acne (35.3%), followed by headache (11.8%) and dyspepsia (11.8%) [78]. Most patients experienced mild adverse events, and no treatment discontinuations due to adverse events were observed [78]. Another selective JAK 1 inhibitor, abrocitinib, was also approved by the KFDA in November 2021 to treat patients with moderate to severe AD aged 12 years and older. Despite the potential for several adverse effects of JAK inhibitors, selective JAK inhibitors have been used to treat patients with severe AD due to their superior clinical efficacy over conventional immunosuppressive agents in Korea. However, there are limitations to using biologics and JAK inhibitors in Korea due to high costs and insurance-related issues, which could limit access for those who could benefit from them. In addition, it is not possible to change from one type of JAK inhibitor to another in Korea, and it is not possible to change to a JAK inhibitor after biologic treatment or to a biologic after JAK inhibitor treatment under the current indications, so further improvements are needed on these points. If these problems are resolved, patients with severe AD in Korea may be able to receive more effective treatment with optimal advanced therapies.

## 7. Discussion

AD is a chronic inflammatory skin condition affecting millions worldwide, including in Korea. AD has a significant impact on QoL, and its prevalence has been increasing in recent years. Through this review, we found that the burden of AD in Korea is substantial. Patients with AD experience significantly poor QoL due to AD, which affects their daily activities and emotional well-being. Despite the high prevalence and significant impact of AD, there are many unmet needs in diagnosing and managing the disease in Korea. Due to its heterogenetic nature and absence of a specific biomarker for AD, diagnosis of AD is typically made based on clinical features, including a history of pruritus, typical morphology and distribution of skin lesions, and a personal or family history of atopic disease. Therefore, some misdiagnoses can occur based on current diagnostic criteria in Korea. In terms of management, there is a need for more effective, safe, and cost-beneficial treatments for AD.

The strength of this paper is that it identifies the disease burden and unmet needs of AD in the Korean population. This paper will provide a better understanding of the current situation in controlling AD in Korea and provide more realistic suggestions for improving unmet needs. 

Our review has some limitations. During the literature search, we only included original articles or review articles published in English or Korean in this review. In addition, this paper only covers the updated literature until 28 February 2023, so it does not cover subsequent updates. 

## 8. Conclusions

AD is a significant burden in Korea, and there are many unmet needs in diagnosing and managing the disease. More research is needed to understand AD pathophysiology better and develop more effective and safe treatments in Korea. In addition, efforts are needed to improve education and awareness of the disease among healthcare providers and the public in order to improve the diagnosis and management of AD in Korea.

## Figures and Tables

**Table 2:** Diagnostic criteria of atopic dermatitis in Korea by Atopic Dermatitis Research Group (2005) [52].

Major Features
1. Pruritus
2. Typical morphology and distribution
(1) Under the age of 2 years: face, trunk, and extensor involvement
(2) Over the age of 2 years: face, neck, and flexural involvement
3. Personal or family history (atopic dermatitis, asthma, allergic rhinitis)
**Minor features**
1. Xerosis
2. Pityriasis alba
3. Periorbital eczema or orbital darkening
4. Periauricular eczema
5. Cheilitis
6. Tendency towards non-specific hand or foot dermatitis
7. Scalp scale
8. Perifollicular accentuation
9. Nipple eczema
10. Itch when sweating
11. White dermographism
12. Skin prick test reactivity
13. Elevated serum IgE
14. Tendency towards cutaneous infections

## Data Availability

Data sharing not applicable.
